# Still Relevant, Still Effective: A Retrospective Observational Cohort Study on Real-Life Use of Flunarizine in Episodic Migraine

**DOI:** 10.3390/brainsci15060545

**Published:** 2025-05-22

**Authors:** Devrimsel Harika Ertem, Faik Ilik, Mustafa Kemal Ilik

**Affiliations:** 1 Department of Neurology, Silivri Anatolian Hospital, 34570 Istanbul, Türkiye; 2 Department of Neurology, Medova Hospital Affiliated with Medical Faculty, KTO Karatay University, 42208 Konya, Türkiye; faikilik@hotmail.com; 3 Department of Neurosurgery, Buyuksehir Hospital, 42060 Konya, Türkiye; mkilik@gmail.com

**Keywords:** flunarizine, headache, migraine, prophylaxis, preventive treatment, observational study

## Abstract

Aim: New disease-specific and mechanism-based treatments for migraine that share good evidence of efficacy have recently been introduced. However, due to reimbursement problems with insurance companies and high costs, classical anti-migraine drugs continue to be used. The objective of this study was to assess the clinical efficacy and tolerability of flunarizine for the preventive treatment of episodic migraine without aura in a Turkish cohort, concentrating on alterations in headache frequency, pain intensity, and migraine-related disability as measured by MIDAS scores within a practical clinical environment. Methods: Clinical and demographic data of 243 patients with episodic migraine without aura (175 females, 68 males; mean age 33.9 years) were evaluated. Headache frequency, side effects of flunarizine, pain intensity, and MIDAS scores were recorded during initial and 3-month follow-up periods. Results: After three months of flunarizine treatment, significant improvements were observed in headache parameters. The mean Numeric Pain Rating Scale (NPRS) score, the mean MIDAS score, and the monthly migraine attack frequency declined significantly (all *p* values < 0.001). Adverse events were reported in 21.8% of patients, most commonly weight gain and tiredness, followed by mood changes, gastrointestinal symptoms, and numbness or tingling. Patients experiencing side effects were significantly older (*p* = 0.023), though side effects did not impact treatment efficacy. Regression analysis identified no significant predictors of disability improvement. Conclusion: Our results demonstrated that flunarizine had considerable short-term efficacy in decreasing the frequency of migraine attacks, alleviating headache severity, and reducing migraine-related disability among patients experiencing episodic migraine without aura. Although mild to moderate side effects were fairly prevalent, especially in older individuals, they did not compromise the effectiveness of the treatment. Notably, early adverse events occurring within the first two weeks resulted in treatment discontinuation for some patients, highlighting the necessity for vigilant monitoring during the initial phase of treatment.

## 1. Introduction

Worldwide, headache disorders impact around 40% of individuals, equating to 3.1 billion people in 2021, with a higher prevalence in females than in males [1]. These disorders rank among the three most prevalent neurological conditions across various age groups, beginning at age 5 and continuing to be among the top three until the age of 80 [1]. In Türkiye, epidemiological studies report a migraine prevalence rate between 10.3% in men and 23.1% in women and a 2.38% incidence rate of migraine, indicating a slightly higher burden compared to global averages [2,3]. Due to the impact of the disease on occupational performance and its effects on social and family life, it is essential to initiate treatment for early episodic and chronic migraines even in the initial stages of the condition. Current treatments officially approved and reimbursed in Türkiye include beta-blockers (e.g., propranolol), tricyclic antidepressants (e.g., amitriptyline), antiepileptics (e.g., topiramate, valproate), and calcium channel blockers (e.g., flunarizine). In recent years, monoclonal antibodies that target the calcitonin gene-related peptide (CGRP) pathway have been introduced as the first mechanism-based and disease-specific preventive therapies for migraine [4,5]. Agents like erenumab, fremanezumab, galcanezumab, and eptinezumab have shown enhanced efficacy and tolerability in comparison to conventional preventive treatments, exhibiting minimal side effects and fostering better patient adherence. A recent review highlighted the expanding spectrum of preventive treatments for migraine, ranging from conventional agents like flunarizine to newer CGRP-targeted therapies, emphasizing the need for accessible and well-tolerated options across different healthcare settings [6]. However, despite their clinical advantages, the substantial cost of these therapies presents a considerable obstacle to their widespread availability, especially in healthcare systems with constrained resources. Although monoclonal antibodies targeting CGRP pathways have become available worldwide, access in Türkiye remains limited due to cost and insurance coverage constraints.

Flunarizine is a non-selective calcium channel blocker with antihistaminergic and dopamine D2 receptor antagonist properties. Flunarizine demonstrates antihistaminergic properties by blocking H1 receptors, which may enhance its sedative and anti-vertiginous effects; however, its primary function in migraine prevention is attributed to its calcium channel blocking capabilities [7]. Its high lipophilicity enables it to cross the blood–brain barrier, where it accumulates in neural tissue [8]. While the precise mechanism in migraine prophylaxis remains unclear, it is hypothesized to act via the inhibition of calcium influx in subcortical structures involved in migraine pathophysiology [9]. Randomized controlled trials have demonstrated its efficacy, comparable to propranolol and topiramate, in reducing migraine frequency and severity [10,11]. In a large UK-based observational study involving 200 patients, flunarizine achieved at least 30% reduction in migraine symptoms in 37% of patients, with a relatively low discontinuation rate due to side effects such as fatigue, mood changes, and weight gain [9].

Despite being listed as a first-line prophylactic agent with Level A evidence in European guidelines [7], flunarizine is not licensed in the United States and remains unavailable in routine clinical practice there, mainly due to concerns about its side effect profile [12]. In the UK, although flunarizine is also not officially licensed, the National Institute for Clinical Excellence (NICE) has issued supportive recommendations for its use in migraine prevention since 2014 [9]. In South Korea, the most commonly used preventive medicine for migraine was reported to be propranolol, followed by flunarizine [13]. Nonetheless, its use is limited due to regulatory barriers, the need for shared-care protocols with primary care physicians, and a lack of familiarity among clinicians [9]. Although flunarizine is considered a first-line prophylactic treatment for migraine in several guidelines, the evidence for its effectiveness, tolerability, and safety in the treatment of episodic migraine is limited [14].

Migraine is a complex neurological disorder involving both peripheral and central mechanisms, including cortical hyperexcitability, thalamo-cortical dysrhythmia, and central sensitization. These pathophysiological features contribute significantly to the burden of disease and highlight the need for mechanism-based preventive approaches [15]. Additionally, migraine is associated with high levels of disability, psychiatric comorbidities, and medication overuse, all of which contribute to poor treatment outcomes [16]. Real-world studies underscore the modest efficacy of traditional preventive drugs, poor adherence, and high dropout rates due to side effects [17]. In an Italian study of migraine patients, the majority experienced less than 50% reduction in headache frequency with preventive treatments. Notably, nearly 20% withdrew from therapy due to adverse effects, and some patients were lost to follow-up, highlighting real-life barriers to sustained treatment [17]. These findings emphasize the urgent need for accessible, effective, and well-tolerated alternatives, especially for patients who often cycle through multiple prophylactic agents without satisfactory relief.

The present study aims to assess the clinical effectiveness and tolerability of flunarizine in Turkish patients with migraine without aura, utilizing retrospective data from a substantial patient cohort. Our evaluation encompasses the analysis of headache frequency, severity, and Migraine Disability Assessments Scale (MIDAS) scores during the administration of flunarizine, following its discontinuation, and throughout a subsequent follow-up period. In light of the limited availability of recent, large-scale data on flunarizine and its ongoing use due to cost-effectiveness and reimbursement policies in Türkiye, this study primarily aims to evaluate the clinical effectiveness of flunarizine in reducing headache frequency, intensity, and migraine-related disability in patients with episodic migraine without aura. The secondary aim is to assess the tolerability profile of flunarizine and explore potential clinical or demographic predictors of treatment response.

## 2. Methods

This retrospective, observational study was conducted in accordance with the ethical principles outlined in the Declaration of Helsinki and was approved by the Ethics Committee of KTO Karatay University, Konya, Türkiye (Study Protocol Number: 2023/011). Informed written consent was obtained from all participating patients.

### 2.1. Study Design and Participants

This study was designed as a retrospective observational cohort study including patients diagnosed with migraine without aura and treated with flunarizine. The study included patients diagnosed with migraine without aura who were admitted to the neurology outpatient clinic of a tertiary care center between 1 May 2022, and 1 May 2024. All subjects had been initiated on flunarizine as part of their migraine treatment regimen. Flunarizine was the first-line treatment in all cases. To minimize clinical heterogeneity and ensure a more uniform study population, all subjects were diagnosed with episodic migraine without aura according to the International Classification of Headache Disorders, 3rd edition (ICHD-3) [18] criteria, as confirmed by a board-certified neurologist specialized in headache disorders.

#### 2.1.1. Inclusion Criteria

- A diagnosis of migraine without aura in accordance with the International Classification of Headache Disorders-3 (ICHD-3) criteria [18].
- Patients aged ≥ 18 years who agreed to participate in the study.
- Patients with complete clinical records.
- At least one follow-up visit three months after the initiation of treatment.

#### 2.1.2. Exclusion Criteria

- Patients with incomplete follow-up data.
- Patients who underwent interventional pain management prior to the current study.
- Patients had additional headache disorders and/or a history of medication-overuse headaches.
- Patients with other neurological disorders.
- Patients with psychiatric diseases and severe brain, liver, cardiac, or renal dysfunction.
- Patients who declined to participate in the research or were lost to follow-up.

**Figure 1:**
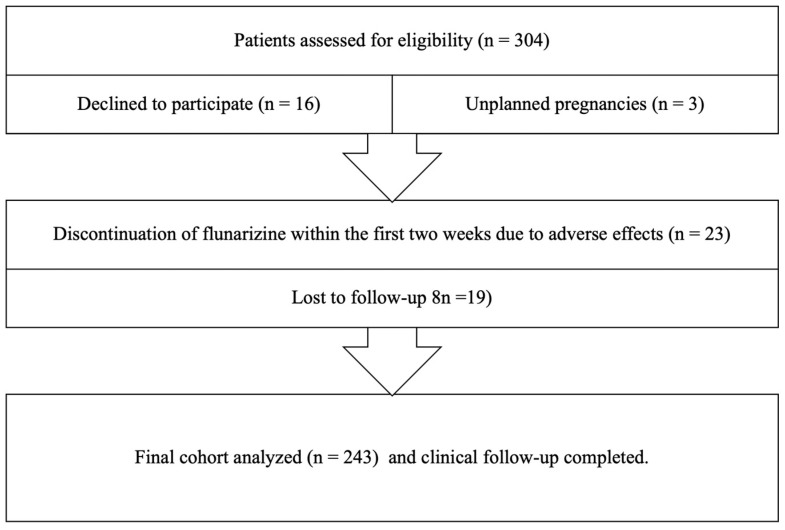
The flow diagram of the study participants.

A total of 304 patients who met the inclusion criteria were enrolled in the study. Sixteen patients, despite meeting the inclusion criteria, declined to participate in the study. Twenty-three patients were excluded from the study due to discontinuation of the medication within the first two weeks because of adverse effects, including tiredness, drowsiness, and severe constipation, respectively. Three women with migraine were withdrawn due to unplanned pregnancies, which prevented them from continuing the treatment. Nineteen patients were lost to follow-up after discontinuing their outpatient clinic visits. As a result, the clinical follow-up was completed for 243 patients. Figure 1 shows the flow diagram of participants in this study.

Demographic data, medical history, presence of comorbidities, number of headache days, and clinical migraine characteristics were systematically extracted. Pain intensity and migraine-related disability were assessed using the Numeric Pain Rating Scale (NPRS) and the Migraine Disability Assessment Scale (MIDAS), respectively, at baseline and at the three-month follow-up visit. Frequently reported adverse effects in the existing literature were compiled, systematically queried during patient follow-up, and documented accordingly. Although standardized headache diaries were not used, subjects were instructed to record the frequency, severity, and duration of migraine episodes, as well as the use of acute medications. These data were retrospectively retrieved from clinical follow-up notes.

### 2.2. Outcome Measures

#### 2.2.1. The Numeric Pain Rating Scale (NPRS)

The NPRS serves as an outcome measure that quantifies pain intensity on a unidimensional scale for adults experiencing chronic pain [19]. Among its various forms, the 11-item NPRS is the most widely utilized. This scale employs an 11-point numeric system, where “0” indicates one end of the pain spectrum (e.g., “no pain”) and “10” signifies the opposite end (e.g., “worst pain imaginable”). The NPRS scores were documented at the initial visit and during a follow-up evaluation after three months to assess the treatment’s efficacy and sustainability in alleviating pain intensity.

#### 2.2.2. Migraine Disability Assessment Scale (MIDAS)

The MIDAS tool is a validated instrument intended to measure headache-related disability experienced over the past three months. It consists of five items that assess the effects of migraines on professional, educational, and household activities. The cumulative score classifies disability into four levels: minimal (0–5), mild (6–10), moderate (11–20), and severe (≥21). In this research, MIDAS was employed to evaluate initial functional impairment and the response to treatment after the use of flunarizine. The validity and reliability of the Turkish Migraine Disability Assessment (MIDAS) questionnaire were assessed by Ertes et al. [20].

#### 2.2.3. Adverse Effect Monitoring

Consistent with the current literature on the side effects of flunarizine, we systematically monitored subjects for frequently reported adverse effects during their follow-up appointments [7,9,12,14,21]. We specifically asked about somnolence, weight gain, mood changes (including symptoms of depression), and extrapyramidal symptoms, as these have been commonly noted in previous research. To facilitate a thorough evaluation, subjects were also posed an open-ended question regarding any additional side effects they may have encountered. This methodology enabled the detection of both expected and new adverse events linked to flunarizine treatment.

### 2.3. Statistical Analysis

All statistical analyses were performed using Python (version 3.11, Python Software Foundation, 2025) in a Jupyter Notebook environment. The pandas, scipy, statsmodels, matplotlib, and seaborn libraries were utilized for data processing, hypothesis testing, modeling, and visualization. Descriptive statistics were computed to summarize demographic and clinical features, with continuous variables expressed as medians and interquartile ranges and categorical variables as frequencies and percentages. The Wilcoxon signed-rank test was used to compare the pre- and post-treatment number of headache days and the NPRS and MIDAS scores. Between-group comparisons were performed using the Mann–Whitney U test for non-normally distributed continuous variables. Associations between age, gender, baseline pain intensity, and MIDAS improvement were further explored using an ordinary least squares (OLS) linear regression model. A two-tailed *p* value of less than 0.05 was considered statistically significant.

## 3. Results

A total of 243 subjects diagnosed with episodic migraine without aura were included in this study, consisting of 175 females and 68 males, with a mean age of 33.9 years. At baseline, the mean NPRS score was 7.19 ± 1.06, which decreased to 4.70 ± 2.04 after three months of flunarizine treatment. A statistically significant reduction in NPRS scores was detected (*p* < 0.00001). The mean MIDAS score was 10.2 ± 5.74 before treatment and declined to 6.88 ± 5.30 at follow-up, showing a significant decline after treatment *p* < 0.00001). The mean monthly migraine attack frequency significantly decreased from 8.45 ± 3.76 at baseline to 3.00 ± 2.19 following flunarizine treatment (*p* < 0.0001).

To examine whether gender influenced treatment outcomes, we compared baseline and post-treatment NPRS and MIDAS scores between male and female subjects. The Mann–Whitney U test revealed no statistically significant differences between the two groups for baseline NPRS (*p* = 0.36), post-treatment NPRS (*p* = 0.51), baseline MIDAS (*p* = 0.92), or post-treatment MIDAS scores (*p* = 0.66). These findings suggest that the clinical effectiveness of flunarizine in reducing pain intensity and migraine-related disability was comparable across genders in this cohort. Table 1 summarizes the research results and statistical methods described above.

**Figure 2:**
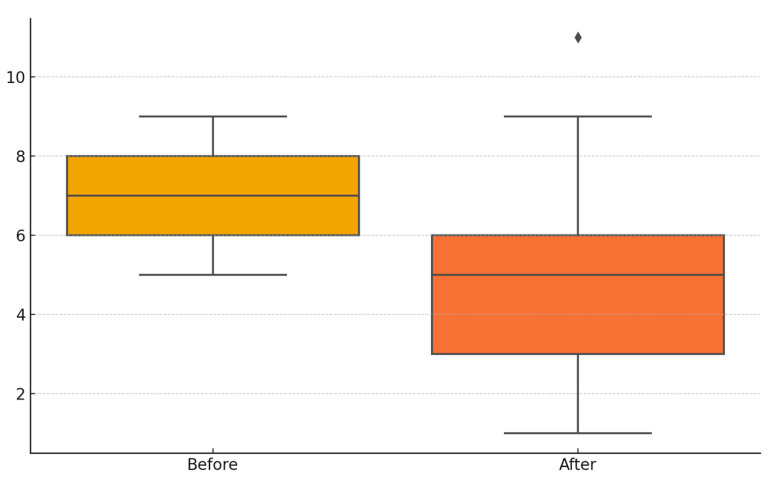
The Numeric Pain Rating Scale (NPRS) scores before and after treatment.

**Figure 3:**
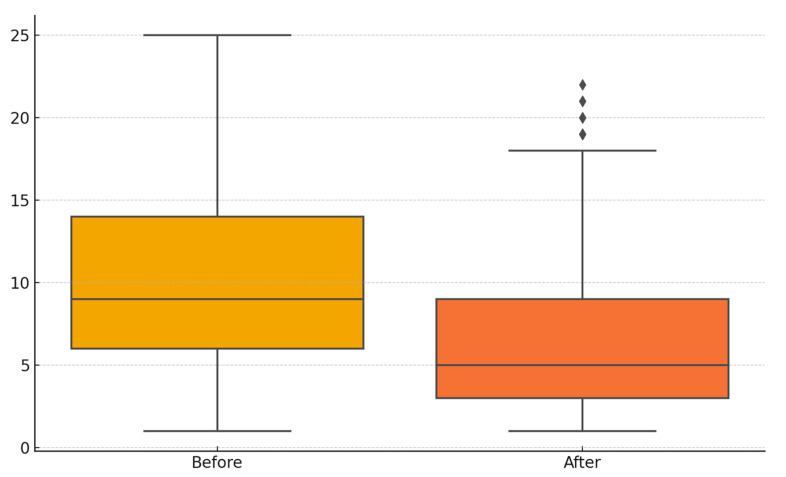
The Migraine Disability Assessment (MIDAS) scores before and after treatment.

Figure 2 and Figure 3 illustrate the mean NPRS and MIDAS scores prior to and following flunarizine treatment.

Among the 243 subjects included in the study, 53 (21.8%) reported adverse effects related to flunarizine use. The most common were weight gain and tiredness, each occurring in 15 subjects (6.2%), followed by mood changes in 10 subjects (4.1%), gastrointestinal symptoms such as nausea or constipation in 8 subjects (3.3%), and numbness or tingling in the hands or feet in 5 subjects (2.1%). When analyzed by gender, tiredness was more frequently reported by women, while weight gain was more common among men.

To explore factors associated with the occurrence of side effects, clinical and demographic characteristics were compared between subjects who experienced adverse events and those who did not. The Mann–Whitney U test showed no significant differences in post-treatment NPRS (*p* = 0.78) or MIDAS scores (*p* = 0.71) between the two groups, indicating that side effects were unrelated to treatment efficacy. However, subjects who reported side effects were significantly older (*p* = 0.023), suggesting that age may play a role in flunarizine tolerability.

A linear regression analysis was conducted to identify predictors of treatment response, using the change in MIDAS score (baseline to 3-month follow-up) as the dependent variable. Independent variables included age, gender, baseline NPRS score, and the presence of side effects. The model did not reveal any significant predictors (R^2^ = 0.008, F(4, 238) = 0.46, *p* = 0.765), and none of the individual variables—age (*p* = 0.583), gender (*p* = 0.692), baseline NPRS (*p* = 0.308), or side effects (*p* = 0.448) were significantly associated with treatment outcome. These findings suggest that reductions in migraine-related disability with flunarizine are not easily predicted by common clinical or demographic factors in this patient group.

## 4. Discussion

This research indicated that flunarizine serves as an effective and generally well-tolerated preventive treatment for patients experiencing episodic migraine without aura in a practical clinical environment. Following three months of treatment, patients reported significant decreases in the frequency and intensity of headaches, as well as in migraine-related disability. It is noteworthy that those with greater initial disability seemed to gain more from the treatment. Although adverse effects were noted in about 20% of patients—predominantly fatigue and weight gain—these side effects did not hinder the effectiveness of the treatment. A portion of patients discontinued treatment within the first two weeks, mainly due to fatigue, drowsiness, and gastrointestinal issues. This highlights the necessity for early monitoring and patient education. Our results endorse the short-term clinical effectiveness of flunarizine, particularly in environments where access to newer migraine-specific therapies is limited.

The use of flunarizine in migraine prophylaxis has been investigated for over four decades. One of the earliest studies by Amery (1983), titled “*A New Prophylactic Drug in Migraine*”, described flunarizine, a calcium channel blocker, as “a safe and effective prophylactic drug for both common and classical migraine”, based on findings from double-blind, placebo-controlled trials and comparisons with other agents such as pizotifen and cinnarizine [22]. Subsequently, in 1984, Frenken and Nuijten published “*Flunarizine, a New Preventive Approach to Migraine*”, reporting the results of a double-blind, placebo-controlled trial in which 17 subjects with common migraine received 10 mg flunarizine daily and 18 subjects received placebo over a 12-week period [23]. Their findings demonstrated a significant reduction in migraine attack frequency in the flunarizine group. In Türkiye, in 1994, Balkan et al. conducted a study evaluating the efficacy of flunarizine in migraine prophylaxis among 35 subjects over a 3-month period, reporting complete elimination of migraine symptoms in approximately one-third of participants [23]. More recent research has shifted focus toward guideline recommendations and theoretical models concerning flunarizine’s role in migraine management [24]. Over the past two decades, flunarizine has often been included in comparative studies assessing the efficacy of newer migraine treatments, frequently serving as a reference agent for evaluating reductions in attack frequency [25,26,27,28,29,30]. In 2018, in a comprehensive study by Karsan et al., the most common indication for flunarizine use was chronic migraine, followed by migraine with aura, sporadic hemiplegic migraine, familial hemiplegic migraine, and new daily persistent headache with migrainous features [9]. The study found flunarizine to be generally effective, although approximately one-quarter of subjects reported no clinical benefit. Our findings regarding the reduction in migraine attack frequency are consistent with previous studies. While most prior studies have primarily focused on migraine attack frequency as the primary outcome, our study contributes to the literature by providing real-world data and evaluating not only attack severity but also migraine-related disability through the use of MIDAS scores. This approach offers a broader perspective on the impact of flunarizine in the management of migraine without aura.

In our research, 21.8% of subjects reported adverse events, with an early discontinuation rate of 9.4% (23 subjects) occurring within the first two weeks of flunarizine treatment due to side effects such as fatigue, drowsiness, and severe constipation. These results are particularly significant considering that most prior studies have mainly concentrated on long-term tolerability. In a follow-up study spanning 24 months involving migraine subjects treated with flunarizine (10 mg daily), drowsiness and weight gain were frequently observed [31]. The incidence of drowsiness was highest during the first month and gradually decreased, showing a notable decline by the conclusion of the treatment. In a study by Colucci D’Amato et al., researchers evaluated the long-term efficacy of flunarizine for migraine prevention, focusing on attack frequency, pain severity, and duration [32]. The most notable improvement was in attack frequency, which decreased by about half within the first three months and remained stable thereafter. Nevertheless, there is a scarcity of data concerning the onset of side effects in the early phases of therapy. The relatively high rate of early adverse events in our study highlights the necessity for careful monitoring during the initial treatment phase. These findings indicate that healthcare providers should be alert for early-onset side effects, which may affect patient adherence even when they are adequately informed about potential risks.

Our research found no notable differences between genders regarding the clinical efficacy of flunarizine for migraine prevention, although minor differences in tolerability were observed. Specifically, fatigue was reported more often by female subjects, while weight gain was more prevalent among male subjects. This observation is consistent with earlier studies indicating that migraines are generally more common and severe in women, influenced by hormonal factors, as noted by Vetvik and MacGregor [33]. Nonetheless, there is a scarcity of data specifically examining gender differences in the response to flunarizine treatment. By demonstrating similar efficacy across genders, our study provides important insights that may guide clinicians in their expectations and dosing practices in everyday clinical settings.

Our analysis revealed that no clinical or demographic variables, including age, gender, initial pain severity, or side effects, were found to be significant predictors of treatment response to flunarizine. This finding contrasts with earlier studies that indicated certain factors could affect migraine treatment results. For example, Bigal et al. [34]. emphasized the impact of psychiatric comorbidities like depression and anxiety on treatment effectiveness, while Lucetti et al. [35]. found that a positive family history of migraine and higher baseline pain intensity was associated with better treatment outcomes, whereas frequent migraine attacks and a history of analgesic overuse were linked to poorer responses. The absence of significant predictive factors in our analysis suggests that flunarizine’s efficacy may be consistent across diverse patient profiles. However, it is important to acknowledge that our study did not assess psychosocial variables, such as depression and anxiety; additionally, subjects with medication-overused headache, which has been implicated in other research as a potential influencer of treatment response, were excluded.

In contrast to large-scale randomized controlled trials (RCTs) that are performed in highly regulated settings, real-world studies offer significant insights into the effectiveness and tolerability of treatments in everyday clinical practice. While RCTs, such as network meta-analysis, have demonstrated the efficacy of various preventive migraine therapies in controlled environments, patient adherence and treatment responses in actual practice may vary considerably [7,36,37]. Our research adds to the expanding collection of real-world evidence by underscoring both the effectiveness and tolerability of flunarizine in a standard care setting. Importantly, we noted early-onset adverse events that led to treatment discontinuation in a portion of subjects, a factor that may be inadequately represented in controlled trials. These results highlight the critical role of real-world data in enhancing the findings of RCTs, providing a more thorough understanding of the performance of preventive migraine treatments beyond controlled settings.

Our research primarily examined the use of flunarizine as a standalone treatment; however, previous studies have highlighted its potential effectiveness when paired with other preventive medications. For example, a randomized clinical trial revealed that the combination of flunarizine and topiramate resulted in superior migraine prevention compared to either medication used individually [11]. Furthermore, another investigation indicated that flunarizine combined with propranolol was more successful in decreasing the frequency of migraines than monotherapy with amitriptyline [38]. Additionally, the supplementary application of flunarizine alongside transcutaneous supraorbital neurostimulation demonstrated enhanced results in migraine prevention [39]. These observations imply that flunarizine could play a significant role in combination therapies aimed at preventing migraines. Nonetheless, the exploration of such combinations was not included in the parameters of our current research.

This research has several limitations that must be taken into account when evaluating the results. Firstly, it was carried out at a single institution, which may restrict the applicability of the findings to wider subject demographics. Moreover, the follow-up duration was relatively brief, concentrating on a three-month treatment period, which may not adequately reflect long-term effectiveness or tolerability outcomes. The study exclusively included patients with episodic migraine without aura; individuals experiencing migraine with aura were not evaluated. These design features may limit the generalizability of our findings to broader migraine populations, including those with more complex comorbidities or different migraine subtypes. Another important limitation is the lack of a placebo control group, which prevents us from distinguishing the specific therapeutic effects of flunarizine from possible placebo-related improvements.

Notwithstanding these limitations, our study exhibits several significant strengths. The substantial sample size bolsters the statistical power and dependability of our results. Additionally, the relatively uniform sociodemographic characteristics of the study cohort lead to more consistent and interpretable findings. A notable strength of this investigation is the identification and documentation of early-onset adverse events, especially those occurring within the initial two weeks of treatment, a timeframe that is frequently underrepresented in existing literature. In summary, the strong patient profile and thorough data collection provide valuable insights into the real-world effectiveness and tolerability of flunarizine for the prevention of episodic migraine without aura.

## 5. Conclusions

This real-world investigation revealed that flunarizine serves as an effective and generally well-tolerated preventive treatment for episodic migraine without aura, leading to a significant decrease in both headache severity and migraine-related disability after three months of therapy. Adverse events, primarily mild to moderate in nature, were reported in approximately 20% of subjects, with older adults showing increased vulnerability; however, these side effects did not compromise the treatment’s effectiveness. A key strength of this research is the early identification of side effects occurring within the initial two weeks, highlighting the importance of vigilant monitoring at the start of treatment. Despite certain limitations, such as being a single-center study, having a short follow-up period, and focusing on a specific migraine subtype, the homogeneity of the patient population contributes to the internal consistency and reliability of the findings. To further validate these findings and explore long-term effects, future multicenter trials with longer follow-up periods, broader inclusion of migraine subtypes, and comparison with a placebo control group are warranted.

## Figures and Tables

**Table 1:** Summary of key statistical tests.

Comparison	Test	*p* Value
NPRS (pre- vs. post-treatment)	Wilcoxon Signed-Rank	<0.00001
MIDAS (pre- vs. post-treatment)	Wilcoxon Signed-Rank	<0.00001
NPRS at 3 months (improved vs. not improved)	Mann–Whitney U	<0.0001
MIDAS at 3 months (improved vs. not improved)	Mann–Whitney U	<0.00001
Migraine attack frequency (pre- vs. post-treatment)	Wilcoxon Signed-Rank	<0.0001
Age (subjects with vs. without side effects)	Mann–Whitney U	0.023
Predictors of MIDAS change (regression model)	OLS Regression	0.765 (ns)

NPRS: Numeric Pain Rating Scale; MIDAS: Migraine Disability Assessment; OLS Regression: Ordinary Least Squares Regression; ns: not significant.

## Data Availability

The raw data supporting the conclusions of this article will be made available by the authors on request.
